# Severe corneal burn due to the accidental application of salicylic acid packed in a plastic dropper bottle

**DOI:** 10.7705/biomedica.5284

**Published:** 2020-06-30

**Authors:** Virgilio Galvis, Alejandro Tello, Néstor I. Carreño, Camilo A. Niño, Natalia A. García, Valeria Otoya, Rodrigo Arana

**Affiliations:** 1 Departamento de Oftalmología, Facultad de Ciencias de la Salud, Universidad Autónoma de Bucaramanga, Floridablanca, Colombia Universidad Autónoma de Bucaramanga Facultad de Ciencias de la Salud Universidad Autónoma de Bucaramanga Floridablanca Colombia; 2 Departamento de Oftalmología, Centro Oftalmológico Virgilio Galvis, Floridablanca, Colombia Centro Oftalmológico Virgilio Galvis Floridablanca Colombia; 3 Departamento de Oftalmología, Fundación Oftalmológica de Santander, FOSCAL, Floridablanca, Colombia Fundación Oftalmológica de Santander FOSCAL Floridablanca Colombia; 4 Facultad de Ciencias de la Salud, Universidad del Rosario, Bogotá, D.C., Colombia Universidad del Rosario Facultad de Ciencias de la Salud Universidad del Rosario BogotáD.C Colombia

**Keywords:** Cornea, corneal opacity, burns, chemical, limbus corneae, epithelium, corneal, Bowman's membrane, córnea, opacidad de la córnea, quemaduras químicas, limbo de la córnea, epitelio anterior, lámina limitante anterior

## Abstract

Eye burns due to the accidental application of pharmacological or nonpharmacological substances packaged in plastic dropper bottles have been described for more than three decades and continue to occur. These burns can cause potentially serious corneal injuries. We report the case of a patient who mistakenly applied salicylic acid to the right eye after confusing it with an eye lubricant, which caused him a severe corneal burn. Fortunately, after aggressive medical and surgical management (including oxygen therapy and amniotic membrane grafting), the visual results were good.

We suggest conducting educational campaigns and taking legislative measures in our country to avoid packaging corrosive substances in this type of dropper bottle to reduce the risk of accidental burns.

## Resumen

Las quemaduras oculares por aplicación accidental de sustancias farmacológicas o no farmacológicas envasadas en frascos goteros plásticos, se han descrito desde hace más de tres décadas y siguen ocurriendo. Estas quemaduras pueden causar lesiones potencialmente graves de la córnea.

Se presenta el caso de un paciente que se aplicó ácido salicílico en el ojo derecho al confundir el envase con el de un lubricante ocular, lo que le causó una grave quemadura corneal. Afortunadamente, tras un manejo médico y quirúrgico agresivo (incluida oxigenoterapia e injerto de membrana amniótica) los resultados visuales fueron buenos. Se sugiere hacer campañas educativas y adoptar normas en el país para evitar el envase de sustancias corrosivas en este tipo de frascos goteros, con el fin de disminuir el riesgo de quemaduras accidentales.

Chemical eye burns constitute a true ophthalmological emergency and are responsible for 1.3% to 6.4% of emergency consultations for ocular causes ^1^. They must be treated immediately and can have a devastating impact on the patient's vision and quality of life. Therefore, the establishment of preventive measures and strategies to reduce the frequency of these accidents in the future is vital.

Approximately two-thirds of eye burns occur with chemicals at work; however, another cause of eye burns that has been described since the 1970s and, unfortunately, continues to occur is when a corrosive element is accidentally applied in the eyes after confusing it with ophthalmic eye drops given the similarity of the bottles containing them. This same mistake can be made by a patient or by health personnel ^2^^-^^13^.

We describe the case of an adult patient who presented an eye burn due to this type of accident.

## Clinical case

A 59-year-old male patient arrived at the emergency department of the *Fundación Oftalmológica de Santander - Foscal* in 2017 after having accidentally applied on his right eye some drops that he used as an antifungal. These drops, manufactured by a laboratory with no approval from the drugs regulatory agency in Colombia (*Instituto Nacional de Vigilancia de Medicamentos y Alimentos*, Invima), apparently contained salicylic acid and were packaged in a plastic dropper bottle. The accident occurred because he confused the bottle with one containing eye lubricant.

Upon admission, three hours after the event, palpebral soft edema, inferior chemosis, and staining in some areas of the bulbar conjunctiva were found in the affected eye. A central abrasion area of approximately 4 x 4 mm was visible in the cornea and the rest of it was covered by a white-grayish membrane of necrotic epithelial tissue. The tarsal conjunctiva was also covered by a similar membrane. Cellularity was found in the anterior chamber (+) and medium mydriasis.

Profuse washing and subtotal removal of the corneal and conjunctival necrotic material were performed (the necrotic tissue was firmly attached to the corneal periphery).

Chemical burn of the ocular surface involving the cornea, limbus, bulbar, and tarsal conjunctiva of the right eye was diagnosed. Topical medications were initiated: 0.3% gatifloxacin + 1% prednisolone (Zypred™, Allergan) every 3 hours, 0.4% sodium hyaluronate (Lagricel™, Sophia) every hour, and 0.5% oxytetracycline + 10,000 IU polymyxin b (Terramicine™ ophthalmic ointment, Pfizer) every 8 hours. Additionally, 1000 mg vitamin C orally every 8 hours and 100 mg doxycycline (Etidoxina™, Euroetika) orally every 12 hours were administered.

**Figure 1:**
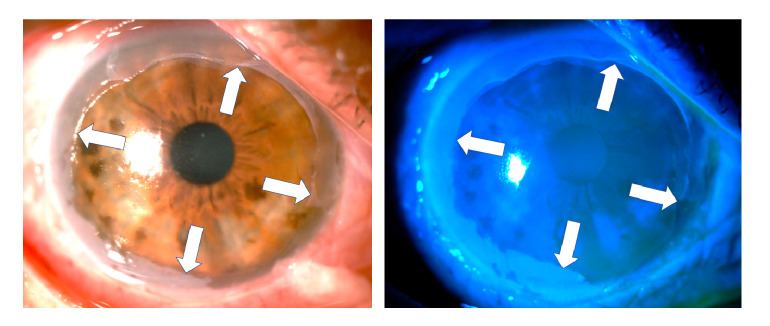
Six hours after the acid burn and after debridement of the necrotic tissue membrane covering the cornea, a remnant could be seen in the entire corneal periphery (white arrows). The de-epithelized area of the cornea is stained very slightly with fluorescein. In the remnant of the peripheral necrotic membrane, obvious fluorescence was observed when using the cobalt blue filter (right).

**Figure 2:**
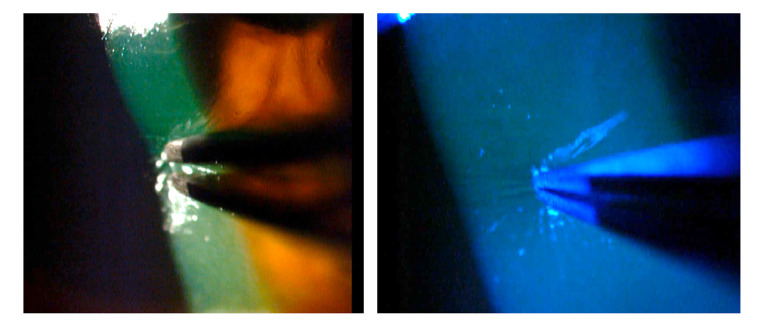
Attempting debridement over the central area of the cornea confirmed the absence of epithelium.

At assessment six hours later, we found almost total de-epithelization of the cornea and a membrane of necrotic tissue 360 degrees around the peripheral cornea and the adjacent limbus. The de-epithelized area showed very little fluorescein uptake (figure 1). The absence of epithelium was confirmed by attempting a debridement of the central area of the cornea with forceps, which evidenced a hardened membrane seemingly corresponding to a Bowman's membrane with changes in protein coagulation (figure 2).

**Figure 3:**
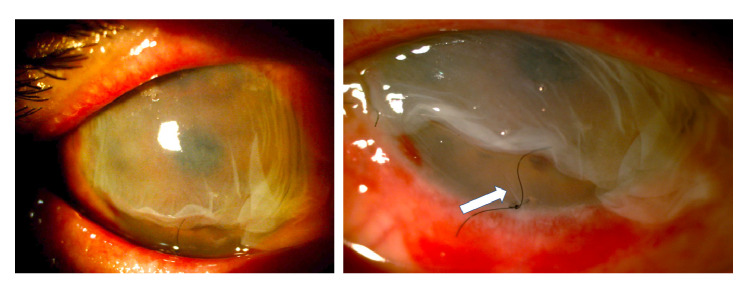
Graft with a double amniotic membrane covering the entire cornea and conjunctiva fixed with sutures and tissue glue and a retaining ring placed in the conjunctival cul-de-sac. Ten days later, the outermost membrane was in partial resorption but the amniotic membrane adjacent to the cornea remained in situ as confirmed by the presence of fine folds adjacent to the suture (white arrow) in the figure on the right.

Given the severity of the condition, an emergency surgical procedure was performed the next day with an amniotic membrane graft covering the entire ocular surface and supported by a retention ring made of intravenous tubing ^14^. On the first postoperative day, the retention ring presented displacement and extrusion with the loss of the membrane. A new graft was performed with a double amniotic membrane fixed with tissue glue (fibrin sealant) and anchored to the membrane using 6-0 polypropylene mattress sutures and bolsters. A total of 0.4 ml of autologous plasma enriched with platelet growth factors, antiangiogenic (12.5 mg bevacizumab) and steroid (20 mg triamcinolone) were also delivered through subconjunctival injection (figure 3).

**Figure 4:**
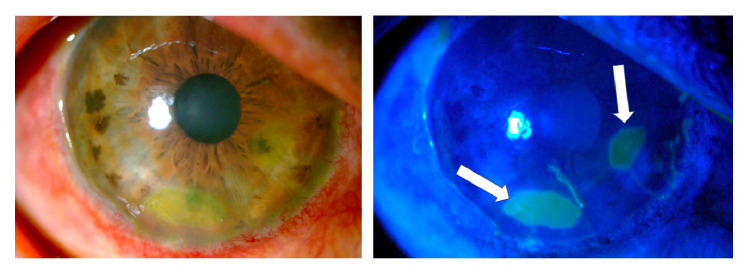
Three weeks after the burn, the amniotic membrane on the surface of the cornea had been reabsorbed and a healthy-looking epithelium was observed with only two oval de- epithelialization areas stained with fluorescein (arrows).

One week after the burn, one-hour daily 100% oxygen therapy via a face mask at 10 L/min was started (16 sessions). Besides, six sessions of 5 L/min of oxygen application were performed on the right eye with a camera made for that purpose ^15^^,^^16^. Twenty days after the burn, the amniotic membrane had been almost completely reabsorbed, with only a remnant observed on the temporal conjunctiva, and 95% of the cornea was epithelialized with only two remaining areas with fluorescein staining (figure 4).

**Figure 5:**
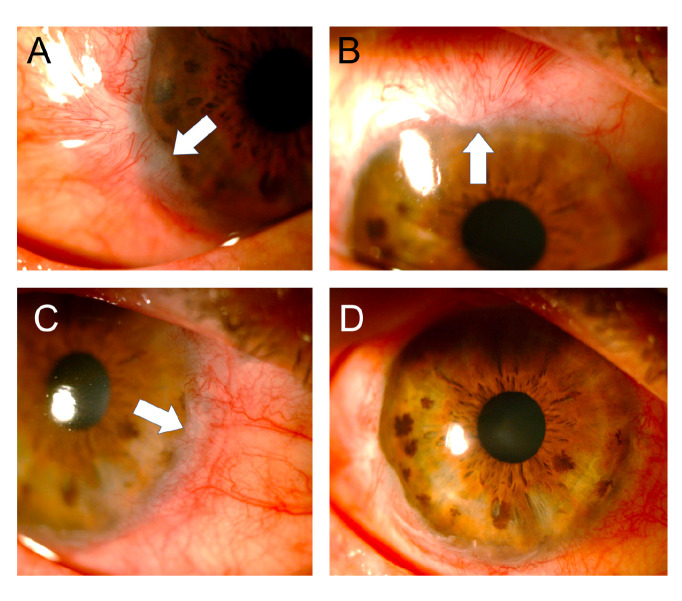
Three months after the accident, conjunctivalization was observed compromising three quadrants of the cornea but with the invasion of less than 2 mm (**A-C:** white arrows). The central cornea showed good transparency (**D**).

After one month, the biconjugate of gatifloxacin and dexamethasone was changed to 0.5% loteprednol (Lotesoft™, Poen) in a two-month taper scheme. On examination, there was a central cornea with haze, inferior punctate keratitis, and mild conjunctivalization between the 9 and 10 o'clock meridians. The corneal haze decreased progressively in the following months reaching good central corneal transparency while the conjunctivalization increased until, by the third month, it compromised three quadrants with the invasion of approximately 1 mm of the peripheral cornea (figure 5).

In March, 2018, (seven months after the burn), the clinical picture was stable and good central corneal transparency (without fluorescein staining) and a slight decrease of conjunctivalization were observed. Preservative-free lubricants were prescribed.

**Figure 6:**
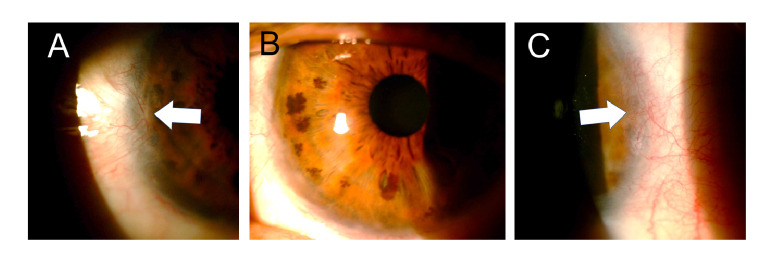
Two years after the burn, the cornea presented good transparency (**B**) and the conjunctival areas only involved two quadrants and showed significant regression (**A and C**).

In the last control, two years after the burn, conjunctivalization was evident only in two quadrants (figure 6). The cornea had good transparency and showed no fluorescein staining. Visual acuity was found in 20/40 without correction and reached 20/20 with + 0.50-0.50 x 90.

## Discussion

Corneal burns can be caused by chemical substances (solid, liquid, dust, or vapor) when they come into contact with the ocular surface. These chemicals can be alkalis or acids contained in several products including detergents, disinfectants, and solvents. Generally, the lesions caused by alkalis (ammonia, magnesium hydroxide, lime, and bleach) are more serious than those caused by acids (sulfuric, hydrochloric, and nitrous) because they have a greater capacity to penetrate tissues ^17^^,^^18^.

Immediate copious irrigation, even using regular tap water, is universally recommended in acute eye burns preferably at the moment of the accident at home or in the workplace. Eye washing should be continued in emergency wards ^18^^,^^19^. Medical therapy involves the use of agents to promote epithelialization, minimize inflammation, and prevent scar complications. Biological fluids including umbilical cord blood serum, amniotic membrane suspension, autologous serum, and autologous plasma rich in growth factors promote healing when used as supplements of conventional therapy ^18^^,^^19^. Surgical treatment of acute eye burns includes debridement of the necrotic tissue, application of tissue adhesives (fibrin glue), tenoplasty, and tectonic keratoplasty. Amniotic membrane transplantation is a surgical treatment frequently used as a complement of the conventional treatment to promote epithelial healing, minimize pain, and restore visual acuity. Although many cases with positive results have been published, clear evidence of its positive effect is scarce ^18^^-^^20^. Another rarely used alternative is systemic or local oxygen therapy as proposed by Sharifipour, *et al.*^15^^,^^16^. In our case, we obtained good results by adding to the medical management both the use of the amniotic membrane and oxygen therapy using masks or cameras over the eye.

A finding that caught our attention was that, initially, the examination did not show a clear uptake of fluorescein on the corneal surface, which was almost completely devoid of epithelium; this was confirmed when we scraped with forceps in the central area and were not able to lift any cell layer that could correspond to the epithelium (figures 2 and 3). This may be explained because due to the coagulation necrosis caused by the acid, the epithelium became a white-grayish membrane that was lost in 90% of the corneal area with only a peripheral remnant, not viable, over 2 mm of peripheral cornea, remaining (figure 1) ^17^. The area thus exposed very possibly corresponded to a Bowman's membrane that, due to the severe changes in its protein coagulation, stained very little with fluorescein. We have seen this in a couple of cases of severe acid burns. To the best of our knowledge, this finding has not been reported, although there is no doubt that those frequently receiving patients with corneal acid burns have seen it. However, someone with little experience can be confounded by this and conclude that there is no corneal epithelial compromise because a clear uptake of fluorescein is not visible. However, there might be a total compromise of the corneal epithelium and, therefore, it is something that should be considered.

Despite having a favorable evolution, our patient secondarily developed partial limbal insufficiency due to the severity of the incident, which fortunately remained stable.

**Figure 7:**
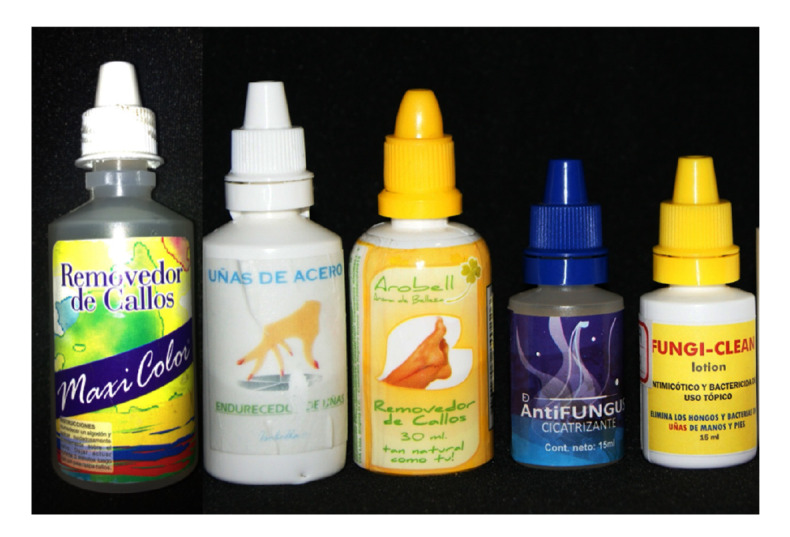
Squeezable plastic dropper bottles containing salicylic acid produced by informal laboratories can be used as keratolytic or antifungal substances for dermatological conditions. However, these bottles can easily be confused with a dropper bottle for ophthalmic medication.

The case we report and other similar ones previously published demonstrate the danger of packaging caustic substances in squeezable dropper bottles that can be mistakenly confused with those containing ophthalmic medications (figure 7). The American Academy of Ophthalmology has a policy statement recommending uniform color-coding of caps and labels for topical eye medications ^21^; surprisingly, to the best of our knowledge, currently there is no standardized policy to avoid packaging dangerous pharmacological or nonpharmacological substances for the eyes in squeezable plastic dropper bottles similar to those used for eye medications. We plan to initiate an awareness campaign in this regard both for the general public through the media and in scientific publications focused on health professionals starting with the present report to engage them in the prevention of these accidents and in advising patients to store medications in completely separate places from nonpharmacological substances in similar containers. Additionally, we will resume the work that we started a few years ago to ensure that the regulations in our country prohibit the packaging of dangerous substances in squeezable plastic dropper bottles that can be confused with ophthalmic eye drops.

Surprisingly, there are many unauthorized presentations of salicylic acid in this type of soft plastic container, as well as several commercial presentations of drugs for dermatological topical use that contain this caustic substance and have been authorized by the Colombian regulatory body (Invima) in this sort of packaging (plastic dropper bottles). Other manufacturers have modified their presentation and do so in glass bottles with brushes or spatulas attached to the lid, which avoids the risk of accidental application on the eyes. If a manufacturer argues that it is essential to pack a hazardous substance in a dropper bottle, then it should be mandatory to use glass bottles and droppers. If plastic must be used, the shape of the lid should be changed (including, for instance, a truncated cylinder-shaped end) so that it is easy to differentiate them from eye drops containers. Additionally, these plastic bottles should have a safety cap with safety locks to avoid child manipulation and patients confusing them with eye medication.

Scientific articles have been suggesting such measures for more than 30 years; however, in many cases they have been ignored and, therefore, we continue to receive cases that are completely preventable ^21^^-^^23^.

## References

[1] Galvis V, Díaz AL, Ochoa ME, Rey JJ, Ardila LC, Olivero LP (2019). Primary causes of emergency ophthalmological consultations at a tertiary care institution in Colombia.

[2] Gudas PP (1979). Ophthaine drug alert.

[3] Ling RF, Villalobos R, Latina M (1988). Inadvertent instillation of hemoccult developer in the eye: Case report. Arch Ophthalmol.

[4] Steinemann TL, Henry KE (1995). Misuse of nonophthalmic and ophthalmic drops due to packaging similarity. Arch Ophthalmol.

[5] Wheeler J, Shah P (2001). Minerva picture. BMJ.

[6] Haylor V, Daines J (2004). Olbas oil mistaken as eye drops. Letter. Pharm J.

[7] Adams MK, Sparrow JM, Jim S, Tole DM (2009). Inadvertent administration of Olbas oil into the eye: A surprisinqly frequent presentation. Eye (Lond).

[8] Shazly TA (2011). Ocular acid burn due to 20% concentrated salicylic acid. Cutan Ocul Toxicol.

[9] Brown JA (2013). Medicinal mishap. Incorrectly dropped in the eye. Australian Prescriber.

[10] Jamison A, Lockinqton D (2016). Ocular chemical injury secondary to electronic ciqarette liquid misuse. JAMA Ophthalmol.

[11] Johnson TV, Pandit RR, Weinberq RS (2017). Ocular chemical burns secondary to unintentional instillation of aqua reqia hobbyist reaqent: Not all that qlitters is qold. JAMA Ophthalmol.

[12] Jinaqal J, Gupta PC, Gupta G, Sahu KK, Ram J (2018). Ocular chemical burns from accidental exposure to topical dermatoloqical medicinal aqent. Indian J Ophthalmol.

[13] Parker RT, McCall DP, Samarawickrama C (2018). Eye injury from toxic chemical mistaken for eye drops. Med J Aust.

[14] Ma KN, Thanos A, Chodosh J, Shah AS, Mantaqos IS (2016). A novel technique for amniotic membrane transplantation in patients with acute Stevens-Johnson syndrome.

[15] Sharifipour F, Baradaran-Rafii A, Idani E, Zamani M, Jabbarpoor-Bonyadi MH (2011). Oxyqen therapy for acute ocular chemical or thermal burns: A pilot study. Am J Ophthalmol.

[16] Sharifipour F, Panahi-Bazaz M, Idani E, Haiizadeh M, Saki A (2015). Oxyqen therapy for corneal edema after cataract surqery. J Cataract Refract Surq.

[17] Yanoff M, Sassani JW (2020). Ocular Patholoqy. Eiqht edition.

[18] Bizrah M, Yusuf A, Ahmad (2019). Eye (Lond).

[19] Sharma N, Kaur M, Aqarwal T, Sanqwan VS, Vaipayee RB (2018). Treatment of acute ocular chemical burns. Surv Ophthalmol.

[20] Clare G, Suleman H, Bunce C, Dua H (2012). Amniotic membrane transplantation for acute ocular burns. Cochrane Database. Syst Rev.

[21] American Academy of Ophthalmology (2019). Color codes for topical ocular medications.

[22] Fraunfelder FT (1988). Drug-packaging standards for eye drop medications. Arch Ophthalmol.

[23] Cohen MR, Davis NM (1992). How to prevent eye medication errors. Am Pharm.

